# A Water-Soluble Thermoplastic Polyamide Acid Sizing Agents for Enhancing Interfacial Properties of Carbon Fibre Reinforced Polyimide Composites

**DOI:** 10.3390/ma17112559

**Published:** 2024-05-26

**Authors:** Chengyu Huang, Peng Zhang, Bo Li, Mingchen Sun, Hansong Liu, Jinsong Sun, Yan Zhao, Jianwen Bao

**Affiliations:** 1 School of Materials Science and Engineering, Beihang University, Beijing 100191, China; huangchengyu@buaa.edu.cn (C.H.); smc703@126.com (M.S.); 2 Composite Technology Center, AVIC Manufacturing Technology Institute, Beijing 100012, China; 050131zhangpeng@163.com (P.Z.); liuhansongzhfc@foxmail.com (H.L.); sunjsbuaa@163.com (J.S.); 3 School of Optics and Photonics, Beijing Institute of Technology, Beijing 100811, China; libo@cimm.com.cn

**Keywords:** carbon fiber, polyimide resin, thermoplastic, water-soluble agent, interfacial performance

## Abstract

Carbon-fiber-reinforced polyimide (PI) resin composites have gained significant attention in the field of continuous-fiber-reinforced polymers, in which the interfacial bonding between carbon fiber and matrix resin has been an important research direction. This study designed and prepared a water-soluble thermoplastic polyamide acid sizing agent to improve the wettability of carbon fiber, enhance the van der Waals forces between carbon fiber and resin and strengthen the chemical bonding between the sizing agent and the alkyne-capped polyimide resin by introducing alkyne-containing functional groups into the sizing agent. This study found that the addition of a sizing layer effectively bridged the large modulus difference between the fiber and resin regions, resulting in the formation of an interfacial layer approximately 85 nm thick. This layer facilitated the transfer of stress from the matrix to the reinforced carbon fiber, leading to a significant improvement in the interfacial properties of the composites. Adjusting the concentration of the sizing agent showed that composites treated with 3% had the best interfacial properties. The interfacial shear strength increased from 82.08 MPa to 108.62 MPa (32.33%) compared to unsized carbon fiber. This research is significant for developing sizing agents suitable for carbon-fiber-reinforced polyimide composites.

## 1. Introduction

Carbon fiber is a high-performance fiber material with a carbon content of more than 95%. About 90% of the high-strength and ultra-high-strength carbon fiber currently used is PAN-based carbon fiber, and the preparation process of PAN-based carbon fiber mainly consists of two stages: the first stage is the preparation of PAN filaments; the second stage is the pre-oxidation and carbonization of PAN filaments, in which the content of carbon atoms increases continuously, and carbon fibers are eventually formed through reactions such as dehydrogenation, cyclisation, cross-linking and polycondensation. The advantages of carbon fiber, such as light weight, high strength, high temperature resistance, electrical conductivity and small coefficient of expansion, have led to a wide range of applications in the fields of aerospace, national defense and military industries, medical devices and rail transportation [1,2,3,4,5,6]. After undergoing high-temperature carbonization and graphitization during the manufacturing process, carbon fiber forms a disordered layer of graphite on its surface. This results in a lack of active functional groups on the surface of the carbon fiber, which in turn causes poor wettability between the carbon fiber and resin, leading to the destruction of the interface area during the preparation of composites [7]. Usually, researchers improve the interfacial compatibility between carbon fiber and resin by modifying the surface of carbon fiber, of which the sizing agent coating method [8,9,10,11,12,13] is the main method of carbon fiber surface modification in the industry.

Carbon fiber surface sizing is an important step in industrial production, which can enhance the agglomeration of carbon fiber and prevent the carbon fiber from being damaged during processing [14]; and the sizing agent simultaneously plays the roles of lubricant and coupling agent [15,16], which effectively improves the wettability of the carbon fiber [17,18,19,20] and enhances the interfacial properties of the composites. Compared to chemical grafting, plasma treatment, electrochemical oxidation, chemical vapor deposition and other modification methods [21,22,23,24,25,26,27], carbon fiber sizing is simple and efficient, with little damage to the mechanical properties of the fiber itself. However, some of the new sizing agents contain strong polar organic solvents. These organic solvents are difficult to remove from the fiber surface and the organic solvent vapor is harmful to the working environment, so the study of water-soluble sizing agents is of great technical importance. Zhang et al. [28] used water-based polyurethane (PU) to size carbon fiber; the results showed that the sizing of PU increased the number of polar functional groups on the surface of the carbon fiber, which led to a decrease in fiber contact angle and an increase in surface energy. Hassan et al. [29] prepared a carbon-nanotube-grafted polyimide (PI-CNT) sizing agent; the wettability, polarity and roughness of the carbon fiber were significantly increased, and the sizing increased the flexural strength, modulus and interlaminar shear strength (ILSS) of the carbon-fiber-reinforced polyetheretherketone composites (CF/PEEK) by 63.2%, 69.9% and 70.5%, respectively, compared to de-sizing the carbon fiber. An easily purified semi-aliphatic polyimide sizing agent was synthesized by Yuan et al. [30]. The sizing agent could be configured as an aqueous solution and resulted in an increase in ILSS and flexural strength of the final composites by 23.82% and 7.8%, respectively, compared to the unmodified fiber-reinforced composites.

Polyimide (PI) is a high-performance polymer with an imide heterocycle in the backbone. Polyimide has excellent thermal, mechanical and dielectric properties, as well as excellent abrasion resistance, flame retardancy, radiation resistance and non-toxicity [31,32,33,34,35]. Traditional matrix resins used in composites, such as epoxy resins and bismaleimide resins, are limited to 150~250 °C, which severely limits the application of composites in high-temperature structures and their components [36,37,38,39]. Polyimide is one of the best high-temperature-resistant matrix materials in resin-based composites, and its multi-generation products can meet the temperature resistance requirement of 300~500 °C, which is currently mainly used in the advanced field of high-temperature-resistant structural components in aircraft [40,41]. The main difference between polyimide composites and traditional composites is that their interface is formed under high-temperature and high-pressure conditions (300~400 °C), which makes the interface formation mode between the polyimide resin matrix and carbon fiber, the interface bond strength, the stress distribution state between the carbon fiber and matrix, etc., significantly different from those of epoxy and bismaleimide resin matrix composites [41,42].

In this work, a water-soluble polyamide acid (PAA) sizing agent was prepared to address the weak interfacial bonding between carbon fiber and the alkyne-capped polyimide resin matrix, and the interfacial properties of the CF/PI composites were improved using this sizing agent. The effects of the sizing agent coating on the chemical components and the physical structure of the carbon fiber surface were quantitatively investigated using analytical test methods such as environmental scanning electron microscopy (SEM), atomic force microscopy (AFM), Fourier-transform infrared spectrometry (FTIR), dynamic contact angle, micro-droplet debonding, nanoindentation and dynamic light scattering (DLS). In addition, the effect of sizing agent concentration on the interfacial properties of alkyne-capped polyimide composites and the mechanism of interfacial layer formation and destruction were also investigated, which provided a data reference for the interfacial improvement and modification method of CF/PI composites, and was of great significance for improving the overall performance of the final polyimide resin matrix composites.

## 2. Materials and Methods

### 2.1. Materials

Carbon fiber CCF800H, 12K, with an average monofilament diameter of 5 μm, was produced by the dry–wet spinning process, provided by Weihai Expand Fiber Co., Ltd. (Weihai, China) Alkyne-capped polyimide resin (PI) was provided by AVIC Manufacturing Technology Institute Composite Technology Center. N,N-dimethylacetamide (DMAc), triethylamine (TEA) and ethylene glycol were purchased from Beijing Hyundai Oriental Chemical Company Limited (Beijing, China). 2,3,3′,4′-BiphenyLtetracarboxylic (α-BPDA), 4,4′-(1,3-phenylenebis(oxy))dianiline (APB) and 3-Ethynylaniline (APA) were purchased from Shanghai Aladdin Biochemical Technology Co., Ltd. (Shanghai, China). All reagents used were analytical-grade and used directly without further treatment.

### 2.2. Preparation of Polyamide Acid Sizing and Sizing Treatment

**Figure 1:**
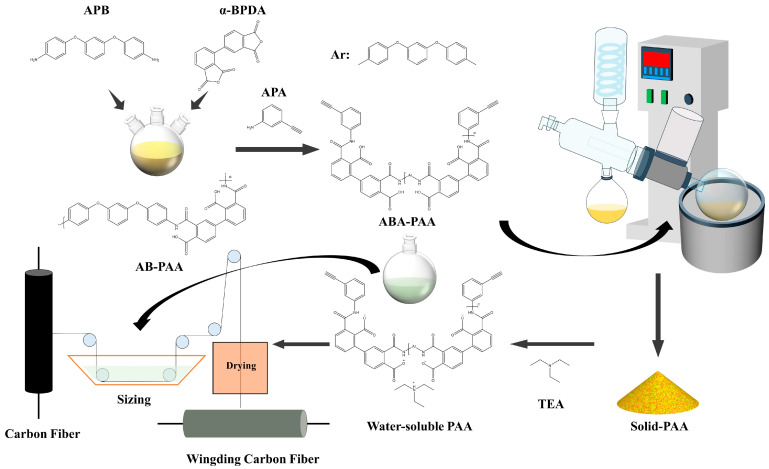
Schematic diagram of the carbon fiber preparation and electrochemical anodic oxidation process of carbon fiber: *: representing APB molecules or α-BPDA molecules at the end of the polymer molecular chain.

As shown in Figure 1, the polymerization degree was designed to be 25 (Figure S1), and APB and α-BPDA were added to the non-protonic solvent DMAc and synthesized at 0 °C under nitrogen atmosphere and mechanical stirring to obtain AB-PAA; then, the molecular weight was controlled by adding the capping agent APA to obtain ABA-PAA. The organic solution obtained was transferred to a rotary evaporator, and the organic solvent was removed at 80 °C to obtain solid PAA sizing agent. After that, TEA was added in deionized water according to the molar ratio of TEA:α-BPDA = 2:1, the solid PAA was mechanically stirred for 10 min after transfer to deionized water containing TEA, and water-soluble PAAs with different concentrations could be obtained after dilution, which were named PAA (1%), PAA (3%) and PAA (5%) according to the concentrations. The obtained water-soluble PAAs were transferred to the sizing tank for continuous process preparation, and finally the solvent was removed using a 100 °C oven to obtain carbon fiber with a sizing layer, named PAA (1%)-carbon, PAA (3%)-carbon and PAA (5%)-carbon, respectively. The unsized carbon fiber was named raw carbon.

### 2.3. Preparation of CF/PI Composites

The composites were prepared from sized carbon fiber with different sizing concentrations and alkyne-based end-capped polyimide resins. Firstly, the prepreg was prepared by combining the polyimide resin film with carbon fiber through continuous production equipment, and then the CF/PI composites were prepared through the lay-up design and the high-temperature and high-pressure molding process in hot press tanks.

### 2.4. Characterization

#### 2.4.1. Scanning Electron Microscopy

The surface morphology of the carbon fiber was observed using a Quanta 450FEG scanning electron microscope (manufactured by FEI Company, Hillsboro, OR, USA). To prepare the specimens for surface morphology observation, carbon fiber tows of approximately 10 mm to 20 mm in length were attached in parallel to the surface of an aluminum sample stage using a conductive adhesive. Scanning electron microscopy (SEM) was also used to analyze the damage profile at the interface of the composites after micro-droplet debonding. The damage profile specimens were prepared by attaching sufficiently tested monofilament specimens to the sample stage surface in parallel with conductive adhesive. All SEM specimens were coated with gold to enhance their electrical conductivity. The SEM test was conducted with an accelerating voltage of 10.0 kV, a working distance of approximately 10 mm and magnifications of 2500 and 5000 times.

#### 2.4.2. Atomic Force Microscopic

The surface morphology and roughness of the carbon fiber were analyzed by using a Dimension ICON (Bruker Technology Co., Ltd., Billerica, MA, USA). AFM specimens were prepared by separating the monofilaments from the carbon fiber tows and attaching the ends of the monofilaments to the slides parallel to each other using epoxy-type adhesive. The AFM tests were carried out in Tapping mode, with a scanning area of 3 μm × 3 μm and a scanning frequency of 1.0 Hz. Roughness was calculated using NanoScope Analysis software 3.0. The interfacial region of the composites was analyzed by using the Atomic Force Quantitative Nanomechanics (AFM-QNM) method at a scanning frequency of 1.0 Hz for a 10 μm × 10 μm surface.

#### 2.4.3. Fourier Transform Infrared Spectrometer

The chemical elements and functional groups on the surface of the carbon fiber were tested using an X-ray photoelectron spectroscopy analyzer, model ESCALab Xi+ (ThermoFisher, Waltham, MA, USA), with 1–2 cm of carbon fiber tows pasted on a special sample stage for the XPS test, and the test was carried out with full-spectrum scanning at 100 eV using Al Kα as the radiation source. The data results were analyzed using XPSPEAK41 software. The C 1s peaks were classified into six peaks: C=C (284.4 eV), C-C (285.5 ± 0.1 eV), C-O (286.5~286.8 eV), C=O (287.6 ± 0.2 eV), C-N (288.0~288.7) and O-C=O (290.0 ± 0.3). Shirley was chosen as the baseline type.

#### 2.4.4. Dynamic Contact Angle Test and Surface Free Energy Calculation

The dynamic contact angle was measured using a DCAT 21 dynamic contact angle meter (Data Physics Instruments, Filderstadt, Germany). Carbon fiber with a reservation length of 6–8 mm was used, with a forward speed of 0.02 mm/s and an immersion depth of 5 mm. Contact angles of the carbon fiber with water and ethylene glycol were tested separately at room temperature, with at least four effective values averaged for each carbon fiber sample. The OWRK method was used to calculate the surface energy, dispersion components and polar components of carbon fiber. The surface energy of deionized water is 72.80 mN/m, with a polar component of 51.00 mN/m and a dispersion component of 21.80 mN/m. The surface energy of ethylene glycol is 47.70 mN/m, with a polar component of 16.80 mN/m and a dispersion component of 30.90 mN/m. The polar and dispersion components of the surface free energy of carbon fiber can be obtained from the following equation:

(1) $$\gamma_{l}(1+cos\theta )=2{\left(\gamma_{s}^{p}\gamma_{l}^{p}\right)}^{1/2}+2{\left(\gamma_{s}^{d}\gamma_{l}^{d}\right)}^{1/2}$$

(2) $$\gamma_{s}=\gamma_{s}^{d}+\gamma_{s}^{p}$$

where $\gamma_{s}$, $\gamma_{s}^{d}$ and $\gamma_{s}^{p}$ are the total surface free energy of carbon fiber, the dispersion component of the total surface free energy and the polar component of the total surface free energy, respectively. $\gamma_{l}$, $\gamma_{l}^{d}$ and $\gamma_{l}^{p}$ are the surface energy, dispersion and polar components of the infiltrated fluid, respectively.

#### 2.4.5. Interfacial Properties of Composite

The interfacial shear properties of polyimide composites reinforced with carbon fiber monofilaments were tested using a micro-droplet debonding tester model MODELHM410, manufactured by Beijing University of Aeronautics and Astronautics. The carbon fiber tow was placed on top of clean release paper. A monofilament was randomly drawn out and pasted on the ‘U’ iron sheet at both ends. Then, a small amount of solution polyimide resin was quickly dipped into the tip of a needle and dotted onto the fiber monofilament. The specimen containing resin microspheres underwent curing in an oven at 135 °C for 30 min, followed by 380 °C for 10 min, before being allowed to cool naturally. After preparing the micro-debonding sample (Figure S2), the micro-debonding test was conducted by suspending the ‘U’-shaped iron sheet on the instrument. Resin microspheres with a size of 20–30 μm were selected as the test object. The instrument was operated to embed the blade into the resin microsphere and the stripping test was performed after fixing it. During the test, the instrument drives the fiber single filament to move upward at a rate of 0.05 mm/min. The instrument records the force–time curve as it observes the position change in the microsphere through the instrument fiber. The interface shear strength can be calculated using the following formula:

(3) $$IFSS=\frac{F_{max}}{\pi dL}$$

where *IFSS* is the interface shear strength, *F_max_* is the maximum force at the resin microsphere stripping moment, *d* is the diameter of the carbon fiber monofilament and *L* is the length of the resin microsphere. Each sample was measured multiple times, and at least eight valid data points were averaged.

#### 2.4.6. Dynamic Light Scattering

The particle size distribution of the water-soluble sizing agent was tested using a laser particle size analyzer model NanoBrook 90Plus (BROOKHAVEN, Ltd., Marlborough, MA, USA) in a test environment of 25 °C for 300 s each time for a total of three tests.

## 3. Results and Discussion

### 3.1. Analysis of Water-Soluble PAA Sizing Agents

**Figure 2:**
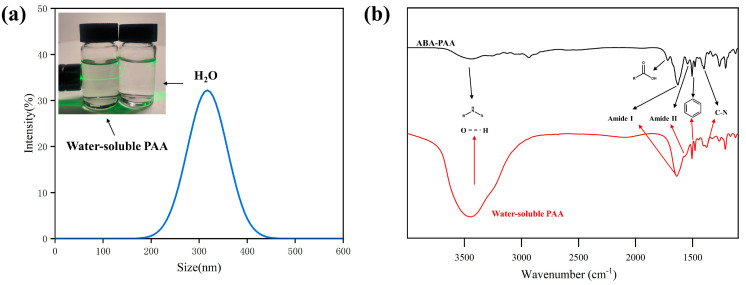
Size distribution curve (**a**) and FTIR spectra (**b**) of PAA sizing agent.

TEA is an alkaline organic reagent that reacts with the acidic carboxyl structure of the PAA molecule, giving TEA a positive charge and PAA a negative charge, both soluble in water. Transparent and stable sizing agents were prepared in deionized water by ionization of solid PAA using alkaline reagents. The Tyndall effect (Figure 2a) was clearly detected by laser irradiation, comparing the water-soluble PAA sizing agent and deionized water, indicating that PAA in aqueous solution is in a colloidal nanoemulsion state. The results of the dynamic light scattering test showed the distribution profile of PAA colloidal particles with an average particle size of 371.3 nm, and the nano particle size contributes to the storage stability of the sizing agent [43]. The zeta potential of water-soluble PAA was −38.1 mV, indicating that the ionic PAA nanoparticles could generate a strong repulsive force between each other, and this repulsive force was sufficient to resist the agglomeration of the particles, thus improving the stability of the sizing agent [44].

The FTIR spectra of the organic solvent PAA sizing agent and the water-soluble PAA sizing agent are shown in Figure 2b. Strong absorption peaks at 1640 cm^−1^, 1506 cm^−1^ and 1410 cm^−1^ were observed for both sizing agents, corresponding to the C=O stretching vibration in the amide Ⅰ, the stretching vibration of the benzene ring skeleton and the C-N stretching vibration in the amide structure; a weaker absorption peak at 1545 cm^−1^ corresponded to the N-H bending vibration of amide Ⅱ [45]. The weak absorption peak at 1545 cm^−1^ corresponds to the N-H bending vibration of amide II, indicating the successful synthesis of the PAA containing amide bonds. After removal of the organic solvent and preparation of water-soluble PAA, the disappearance of the C=O functional group in the carboxylate structure corresponding to the position at 1727 cm^−1^ indicated the complete ionization of PAA, and a strong and broad absorption peak at 3450 cm^−1^ indicated the existence of a large number of intermolecular hydrogen bonds in the water-soluble PAA sizing agent, which were conducive to the infiltration of the sizing agent onto the inert carbon fiber and the bonding with the resin matrix.

### 3.2. Carbon Fiber Surface Morphology and Infiltration Analysis

**Figure 3:**
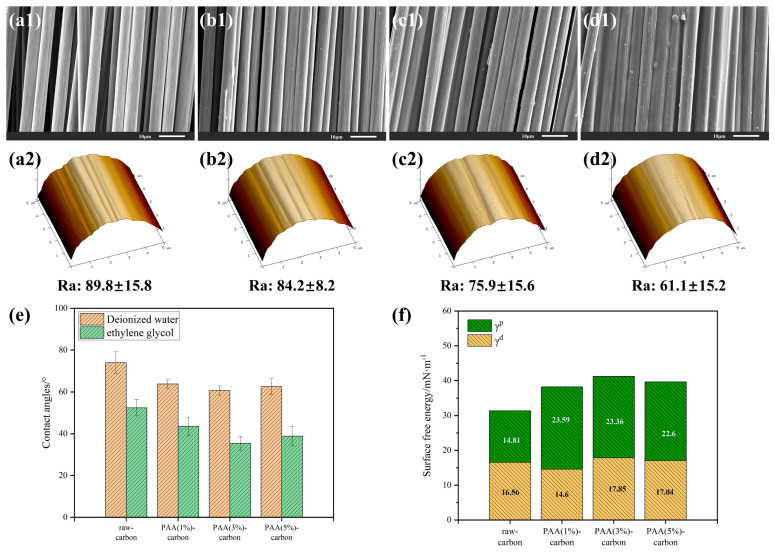
Analysis of carbon fiber surface microscopic morphology and infiltration properties: (**a1**,**a2**) raw−CF; (**b1**,**b2**) PAA (1%)−CF; (**c1**,**c2**) PAA (3%)−CF; (**d1**,**d2**) PAA (5%)−CF; (**e**) contact angle; (**f**) surface free energy.

The surface morphology and roughness of the carbon fiber after sizing with water-soluble PAA were characterized by SEM and AFM, and the elemental carbon and oxygen contents on the carbon fiber surface were examined by energy-dispersive spectroscopy (EDS), as shown in Figure 3 and Table 1. The surface of the unsized carbon fiber (a1) showed typical longitudinal grooves formed by the PAN precursor during the wet spinning of the carbon fiber. After sizing with a 1% concentration of water-soluble PAA, PAA (1%)-CF (b1) still retained the obvious groove structure on the surface and the surface roughness was slightly reduced, indicating that the low concentration of sizing agent did not have a significant effect on the sizing of carbon fiber, making the sizing layer thin and discontinuous. After increasing the concentration of the sizing agent, slightly shallower carbon fiber grooves were observed on the surface of PAA (3%)-CF (c1), and the surface roughness (Ra) decreased from 89.8 nm to 75.9 nm, indicating that the sizing agent was well impregnated and distributed on the carbon fiber surface, and the “ridges” of the carbon fiber grooves on the surface were filled by the film-like sizing agent. The carbon fibers are evenly enveloped by the sizing agent and the normal gaps between the fibers are maintained, which is conducive to subsequent processing and forming. After the sizing agent concentration was further increased to 5%, the sizing agent accumulated on the surface of PAA (5%)-CF (d1), which made it difficult to spread evenly, and the individual fibers stuck to each other; then, the surface of the fiber was relatively smooth and flat, and the roughness was further reduced to 61.1 nm.

A good wettability of the fiber surface is a prerequisite for the formation of a strong interface of composites, and the wettability of the carbon fiber is affected by both the physical roughness and the chemical functional groups of the surface; the dynamic contact angle test of the carbon fiber is shown in Figure 3e,f. The EDS results of the unsized carbon fiber showed that the oxygen content was 2.32%, with fewer oxygen-containing functional groups on the surface; the contact angle with water was 73.92° and that with ethylene glycol was 52.42°, with a surface free energy of 31.37 mN/m, reflecting the surface inertness of the unsized carbon fiber. After sizing with water-soluble PAA, the contact angles of the carbon fiber with the tested liquids were reduced to different degrees, indicating that the introduction of active functional groups on the surface of the carbon fiber improved the wettability between the fiber and the liquid. Among them, the contact angle of PAA (3%)-CF with deionized water decreased to 60.65°; the contact angle with ethylene glycol decreased to 35.39°; the free energy of the fiber surface increased to 41.21 mN/m, which is an increase of up to 31.4%; and the improvement in the fiber surface wettability was the most obvious. In addition, the contact angles of PAA (1%)-CF and PAA (5%)-CF in deionized water and ethylene glycol were also lower than those of unsized carbon fiber, resulting in increases in the surface free energy of 38.19 mn/m and 39.64 mn/m, respectively. It can be seen that the presence or absence of the sizing agent has a significant effect on the surface free energy of fiber, while the change range of the contact angle and surface free energy is small under the conditions of different concentrations of sizing agent. It was also found that the surface free energy increased and then decreased with the increase in sizing agent concentration, which can be attributed to the fact that the carbon fibers were adhered together by the excessive coating of the sizing agent, resulting in the decrease in the specific surface area in the process of wetting with liquid. The increase in surface free energy is attributed to the contribution of polar groups [46] in the sizing layer to the polar component after coating with the sizing agent, and the introduction of oxygen and nitrogen groups provides good adsorption to the polyimide resin, which also provides stronger chemical bonding for interfacial adhesion, and effectively eliminates voids, cracks and other defects in the interfacial region during the composite molding process. The coating with the sizing agent will not damage the mechanical properties of the fiber itself, but will help the fiber and resin to closely combine and spread the load on the fiber, all of which are conducive to the interfacial properties and overall performance of the composites.

### 3.3. Interfacial Properties of CF/PI Micro-Composites

**Figure 4:**
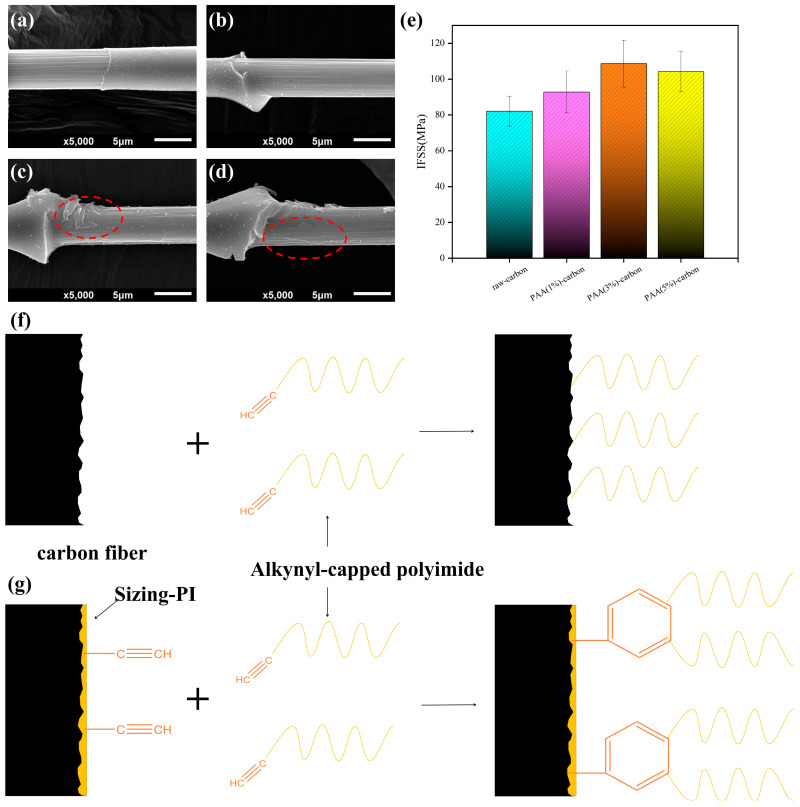
The surface morphology of CFs after PI debonding: (**a**) raw-CF; (**b**) PAA (1%)-CF; (**c**) PAA (3%)-CF; (**d**) PAA (5%)-CF; and (**e**) IFSS of CF/PI and (**f**,**g**) schematic diagram of the reaction mechanism of the interfacial layer of CF/PI composites.

The micro-droplet debonding method was used to evaluate the interfacial properties of the carbon fiber with the alkyne-capped polyimide resin matrix, as shown in Figure 4. The morphology of the carbon fiber surface after the debonding of the resin microspheres was characterized by SEM, and the grooves on the fiber surface of unsized carbon fiber-polyimide composites (Figure 4a) were clear after shear damage, with no obvious resin residue, indicating that cracks in the resin expand rapidly in the interfacial layer during the exfoliation process, leading to rapid slippage of the microspheres and then a lower interfacial shear strength, with an IFSS of 82.08 MPa. In contrast, the interfacial micromorphology of the carbon fiber composites changed significantly after water-soluble PAA sizing, with resin particles sporadically distributed in the interfacial region of the PAA (1%)-CF composites (Figure 4b). When the concentration of sizing agent on the carbon fiber surface was increased to 3% and 5%, more polyimide resin fragments remained on the fiber surface after the resin microspheres were detached (Figure 4c,d), indicating that the bonding part between the sizing agent and the polyimide resin was broken during the crack extension process, which effectively transferred the load from the resin to the fiber, resulting in a higher interfacial shear strength, with the PAA (3%)-CF composites with the best interfacial performance increasing the IFSS to 108.62 MPa (32.33%), as shown in Table 2. In addition, the IFSS of PAA (1%)-CF and PAA (5%)-CF also increased to 92.81 MPa and 104.10 MPa, respectively. From the microscopic morphology of the microspheres after exfoliation, it is concluded that the interfacial damage of CF/PI composites after sizing is mainly of two types: damage at the bond between the carbon fiber and sizing agent and damage at the bond between the sizing agent and resin matrix. The simultaneous occurrence of these two types of damage results in the retention of some resin on the surface of the carbon fiber after the microspheres have been removed, which also effectively increases the interfacial shear strength of the composites.

Based on the above results, the interfacial reinforcement mechanism of carbon-fiber-reinforced polyimide composites is proposed. For unsized carbon fiber (Figure 4f), there are a certain number of surface defects on the surface of the carbon fibers themselves, and the surface chemical activity is low, which results in the formation of fewer chemical bonds when forming the interface of the polyimide composites, leading to poor interfacial bonding between the carbon fibers and their surrounding matrix. The introduction of a water-soluble PAA sizing agent (Figure 4g) allowed the fiber to bond with the polyimide resin through strong chemical bonding, and the amide functional groups contained in the sizing agent reacted with the amide bonds of the polyimide resin during the molding of the composites, resulting in the diffusion and entanglement of the thermoplastic polyimide chain segments at the interface between the sizing agent and the resin. In addition, the alkyne-containing functional groups introduced by the sizing agent can also participate in the heat self-curing reaction of alkyne-terminated polyimide resins to increase the degree of cure of the resins, which is helpful for an effective transfer of interfacial loads, a rapid elimination of stress concentrations and a high dissipation of fracture energy.

**Figure 5:**
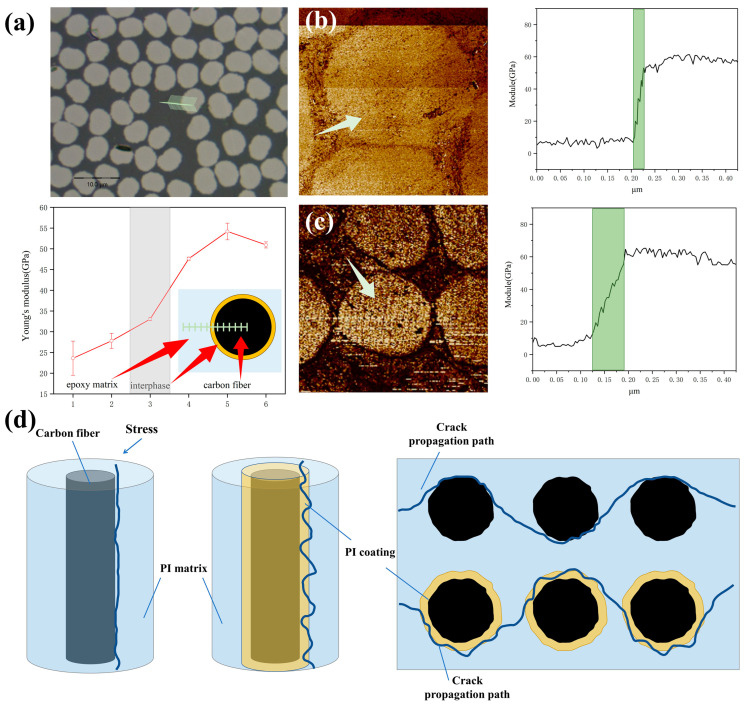
Analysis of interfacial layers of composites: (**a**) nanoindentation test; (**b**,**c**) AFM-QNM test and schematic diagram of interfacial failure mechanism; (**d**) schematic diagram of crack extension in composite.

Based on the above results, the sizing layer formed on the fiber surface by the introduction of the sizing agent is considered to be the key to improving the interfacial properties of composites. The carbon fibers were fixed in a circular rubber box, and then the epoxy resin with a curing agent added was introduced into the rubber box and allowed to cure at room temperature for 24 h. The cured specimens were tested for continuity modulus in the area between the fiber and resin after being fully polished with sandpaper and an automatic polishing machine. The nanoindentation results (Figure 5a) show a small region of modulus increase between the high modulus (carbon fiber) and the low modulus (resin). The results of the AFM-QNM test show that there is a significant difference in modulus between the unsized carbon fiber (Figure 5b) and the resin, whereas there is a transition zone of modulus change between the sized carbon fiber (Figure 5c) and the resin. A slow increase in modulus in this region confirms that there is an interfacial phase consisting of the sizing agent between the fiber and the resin, and the thickness of this interfacial layer is about 85 nm from the modulus change analysis. The contribution of the interface layer to the interfacial properties of the composite material is shown in Figure 5d. For the unsized CF/PI composites, the carbon fibers are in direct contact with the resin; thus, the low chemical inertia of the carbon fiber makes the interfacial adhesion between the carbon fiber and the resin very weak, leading to a relatively low interfacial modulus. When the composites are broken, the stress is not uniformly transferred to each fiber and cracks easily propagate along the weak interface. After size treatment, there is a chemical bond and mechanical interlock between the size and the carbon fiber and resin. The cracks will pass through the sizing layer with strong modulus as they propagate along the interface so that the stresses are dispersed and absorbed in the interfacial region and the interfacial properties of the CF/PI composites are positively affected.

## 4. Conclusions

In this paper, a thermoplastic water-soluble PAA sizing agent was successfully synthesized and prepared to improve the surface inertness of carbon fiber to enhance the interfacial properties of carbon-fiber-reinforced alkyne-sealed polyimide composites. The sizing agent was a colloidal nanoemulsion in aqueous solution with an average particle size of 371.3 nm, and a continuous and uniform PAA sizing layer was formed on the surface of the carbon fiber after sizing treatment, which made the surface grooves of the carbon fiber shallower and the surface free energy increase. By designing the chemical structure of the sizing agent, the introduced alkyne group can participate in the curing process of the polyimide resin, which plays the role of a bridge on the surface of the carbon fiber and improves the permeability and interfacial adhesion between the carbon fiber and the matrix through chemical bonding with the resin matrix. By adjusting the concentration of the water-soluble PAA sizing agent, it was found that the carbon fiber sized with a sizing agent at a concentration of 3% showed the greatest improvement in the interfacial properties of CF/PI composites, from 82.08 MPa to 108.62 MPa (32.33%), which was attributed to the fact that the sizing agent formed an interfacial layer of a certain thickness and modulus between the carbon fiber and the polyimide resin. The interfacial layer alleviates brittle damage between the fiber and the resin due to the sudden drop in modulus when the interface of the composites is damaged, reduces the stress concentration at the interface of the composites and dissipates the crack diffusion energy, which is conducive to the stress transfer of the composites under load, and thus improves the interfacial bond strength of the composites. This research provides a feasible water-soluble sizing agent solution for a good match between carbon fiber and polyimide resins, which is a promising strategy for improving the interfacial properties of carbon-fiber-reinforced alkyne-capped polyimide composites. This also provides a technical reference for the preparation of low-cost and environmentally friendly high-temperature-resistant polyimide composite materials.

## Figures and Tables

**Table 1:** Surface elemental content of carbon fiber.

Carbon Fiber	Atomic Element Content (%)	
	C	O
raw carbon	97.68	2.32
PAA (1%)-carbon	95.58	4.42
PAA (3%)-carbon	90.32	9.68
PAA (5%)-carbon	87.24	12.76

**Table 2:** IFSS of carbon fiber.

Carbon Fiber	IFSS (MPa)
raw carbon	82.08 ± 8.44
PAA (1%)-carbon	92.81 ± 11.73
PAA (3%)-carbon	108.62 ± 13.12
PAA (5%)-carbon	104.10 ± 11.15

## Data Availability

The data presented in this study are available upon request from the corresponding author. The data are not publicly available due to the fact that they are also part of an ongoing study.

## Supplementary Materials

The following supporting information can be downloaded at: https://www.mdpi.com/article/10.3390/ma17112559/s1, Figure S1: Molecular weights of PAA sizing agents; Figure S2: Schematic diagram of the micro-droplet debonding method.
