# Supplementary material for: A Water-Soluble Thermoplastic Polyamide Acid Sizing Agents for Enhancing Interfacial Properties of Carbon Fibre Reinforced Polyimide Composites
Source: Materials (Basel). 2024 May 26;17(11):2559. doi: 10.3390/ma17112559 (PMC11173954; doi:10.3390/ma17112559)
Supplement: Supplementary file 1 [file materials-17-02559-s001.zip › materials-2983064-supplementary.pdf]

A Water-soluble Thermoplastic Polyamide Acid Sizing Agents for Enhancing Interfacial Properties of CF/PI Composites

Chengyu Huang [1], Peng Zhang [3], Bo Li [2], Mingchen Sun [1], Hansong Liu [3], Jinsong Sun [3], Yan Zhao [1,*], and Jianwen Bao [3,*]

Supporting information

Molecular weights of PAA sizing agents were tested by gel permeation chromatography, as shown in Figure S1.

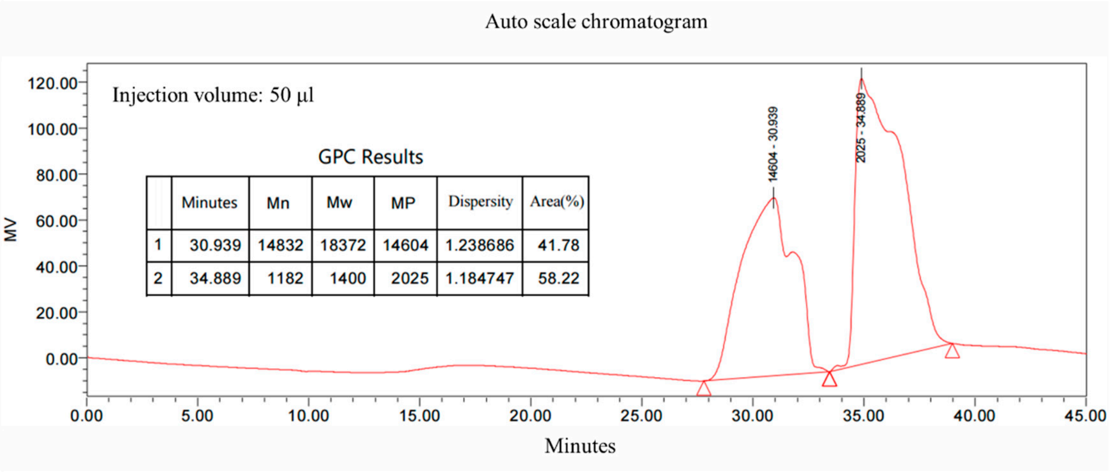

Figure S1. Molecular weights of PAA sizing agents

Schematic diagram of the micro-droplet debonding method, as shown in Figure S2.

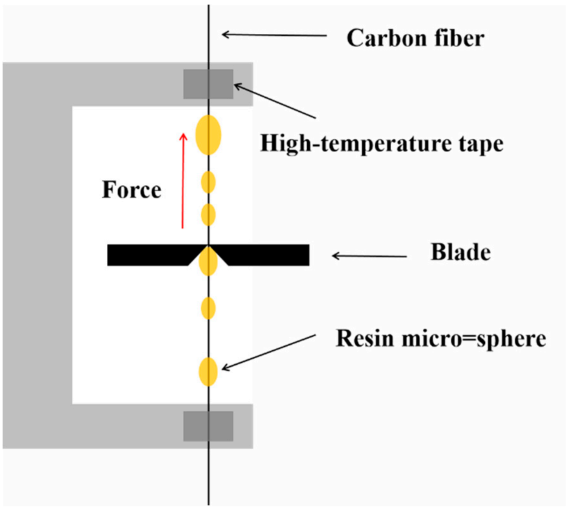

Figure S2. Schematic diagram of the micro-droplet debonding method.
